# A new *Neoplatyura* Malloch from Finland (Diptera, Keroplatidae)

**DOI:** 10.3897/BDJ.2.e1323

**Published:** 2014-09-09

**Authors:** Jukka Salmela, Anna Suuronen

**Affiliations:** † Natural Heritage Services (Metsähallitus), Rovaniemi, Finland; ‡ Zoological Museum, University of Turku, Turku, Finland; § University of Jyväskylä, Dept. of Biological and Environmental Sciences, Jyväskylä, Finland; | Jyväskylä University Museum, Vesilinna, Jyväskylä, Finland

**Keywords:** Fungus gnats, Finland, Lapland, Boreal zone, mires

## Abstract

The genus *Neoplatyura* Malloch is globally represented by 50 species, of which four are European species. In this article a new European *Neoplatyura* from Finland is described. The new species, *Neoplatyura noorae* Salmela, sp. n. is a dark brown species with tibial bristles arranged in rows. The new species is here reported from seven localities in Finnish Lapland. Based on available data, the new species occurs in mires, especially in calcareous rich fens.

## Introduction

The fungus gnat group (Diptera, Sciaroidea excluding Sciaridae) is a species rich nematoceran group belonging to the infraorder Bibionomorpha (Søli et al. 2000, Wiegmann et al. 2011). In Europe, fungus gnats seems to display an anomalous richness gradient being most diverse in the northern boreal region (e.g. Kjaerandsen and Jordal 2007, Kjaerandsen et al. 2007). No less than over 900 species occur in Fennoscandia, of which 768 have been reported from Finland (Kjaerandsen 2012, Jakovlev et al. 2014). Fungus gnats are mostly forest-dwelling insects (Søli et al. 2000), but some species are are usually found on wetlands (e.g. Jakovlev et al. 2014 and references therein). However, the fungus gnat fauna of mires and other wetlands is still poorly known in Fennoscandia, despite the fact that wetlands may cover extensive ares (30–70% of the land area) in the northern parts of the region. In this article we describe a new keroplatid fungus gnat species belonging to the genus *Neoplatyura* Malloch from Finnish Lapland that seems to occur exclusively in mires.

The genus *Neoplatyura* Malloch, 1928 (Malloch 1928) belongs to the family Keroplatidae and its subfamily Keroplatinae (tribe Orfeliini). There are 50 described *Neoplatyura* species, of which 20 are Australasian, 11 Palaearctic, 10 Neotropical, 5 Afrotropical, 2 Oriental and 2 Nearctic (Evenhuis 2006). So far, none of the species has a Holarctic range and four of the Palaearctic species are present in Europe. Following Uesugi (Uesugi 2002), species of the genus can be distinguished by the following morphological characteristics: branches of medial and cubital veins setulose dorsally; prothoracic spiracle with a row of erect black setae on posterior margin; mediotergite and laterotergite bare. However, there is a wide range of genital structure in species assigned to this genus worldwide and it is probably paraphyletic (P.J. Chandler, pers. comm.). Immature stages of the genus are poorly known, but larvae might be predatory, living in soil, or in fungal sporocarps or in decaying wood (Falk and Chandler 2005, Jakovlev 2011).

## Materials and methods

All studied specimens were obtained from Malaise trap samples. The Malaise trap model used was made of cloth with black sides and white cover, (length 110, height 140, width 70 cm) and is suitable for collecting low-flying insects, such as many dipterans. The traps were set in the beginning of June and removed from the field in the late August – mid September; collecting jars were emptied at roughly one month intervals. A solution of 50% ethylene glycol + few drops of detergent was used as a preservative in the traps. The collected material was stored in 70% ethanol. The fungus gnats were sorted from the material in the laboratory. The morphological terminology used here mainly follows Søli et al. 2000. Terminology of some special parts of male genitalia is explained in the figures. The following acronyms for museums and collections are used in the text: MZHF – Finnish Museum of Natural History (Zoological Museum), University of Helsinki, Helsinki, Finland; ZMUT – Zoological Museum, University of Turku, Turku, Finland; JES – Private collection of Jukka Salmela, Rovaniemi, Finland.

## Taxon treatments

### Neoplatyura noorae

Salmela sp. n.

urn:lsid:zoobank.org:act:7EE29C25-5FD1-4E15-A0D4-A555358E2DBE

#### Materials

- **Type status:** Holotype. **Occurrence:** recordedBy: J. Salmela; individualCount: 1; sex: male; **Taxon:** genus: Neoplatyura; specificEpithet: noorae; scientificNameAuthorship: Salmela; **Location:** country: Finland; stateProvince: Lapponia kemensis pars occidentalis; verbatimLocality: Kittilä, Taljavaaranvuoma; verbatimLatitude: 67.579; verbatimLongitude: 25.365; verbatimCoordinateSystem: decimal degrees; verbatimSRS: WGS84; **Event:** samplingProtocol: Malaise trap; eventDate: 2007-6-25/7-24; habitat: rich flark fen; **Record Level:** institutionCode: ZMUT
- **Type status:** Paratype. **Occurrence:** recordedBy: J. Salmela; individualCount: 1; sex: male; **Taxon:** genus: Neoplatyura; specificEpithet: noorae; scientificNameAuthorship: Salmela; **Location:** country: Finland; stateProvince: Lapponia kemensis pars occidentalis; verbatimLocality: Kittilä, Taljavaaranvuoma; verbatimLatitude: 67.579; verbatimLongitude: 25.365; verbatimCoordinateSystem: decimal degrees; verbatimSRS: WGS84; **Event:** samplingProtocol: Malaise trap; eventDate: 2007-6-25/7-24; habitat: rich flark fen; **Record Level:** institutionCode: ZMUT
- **Type status:** Paratype. **Occurrence:** catalogNumber: MYCE-NV-2013-0036; recordedBy: J. Salmela; individualCount: 1; sex: male; **Taxon:** genus: Neoplatyura; specificEpithet: noorae; scientificNameAuthorship: Salmela; **Location:** country: Finland; stateProvince: Lapponia kemensis pars occidentalis; verbatimLocality: Kittilä, Taljavaaranvuoma; verbatimLatitude: 67.579; verbatimLongitude: 25.365; verbatimCoordinateSystem: decimal degrees; verbatimSRS: WGS84; **Event:** samplingProtocol: Malaise trap; eventDate: 2007-6-25/7-24; habitat: rich flark fen; **Record Level:** institutionCode: JES
- **Type status:** Paratype. **Occurrence:** catalogNumber: MYCE-JS-2013-0220; recordedBy: J. Salmela; individualCount: 1; sex: male; **Taxon:** genus: Neoplatyura; specificEpithet: noorae; scientificNameAuthorship: Salmela; **Location:** country: Finland; stateProvince: Ostrobothnia borealis pars borealis; verbatimLocality: Tornio, Rakanjänkkä; verbatimLatitude: 65.890; verbatimLongitude: 24.317; verbatimCoordinateSystem: decimal degrees; verbatimSRS: WGS84; **Event:** samplingProtocol: Malaise trap; eventDate: 2012-7-2/8-6; habitat: rich spring fen; **Record Level:** institutionCode: JES
- **Type status:** Paratype. **Occurrence:** catalogNumber: DIPT-JS-2014-0221; recordedBy: J. Salmela; individualCount: 1; sex: male; **Taxon:** genus: Neoplatyura; specificEpithet: noorae; scientificNameAuthorship: Salmela; **Location:** country: Finland; stateProvince: Lapponia kemensis pars orientalis; verbatimLocality: Sodankylä, Heinäaapa; verbatimLatitude: 67.596; verbatimLongitude: 26.883; verbatimCoordinateSystem: decimal degrees; verbatimSRS: WGS84; **Event:** samplingProtocol: Malaise trap; eventDate: 2012-7-6/8-10; habitat: rich spring fen; **Record Level:** institutionCode: JES
- **Type status:** Paratype. **Occurrence:** catalogNumber: MYCE-NV-2013-0065; recordedBy: J. Salmela; individualCount: 1; sex: male; **Taxon:** genus: Neoplatyura; specificEpithet: noorae; scientificNameAuthorship: Salmela; **Location:** country: Finland; stateProvince: Lapponia kemensis pars occidentalis; verbatimLocality: Kittilä, Silmäsvuoma; verbatimLatitude: 67.582; verbatimLongitude: 25.543; verbatimCoordinateSystem: decimal degrees; verbatimSRS: WGS84; **Event:** samplingProtocol: Malaise trap; eventDate: 2007-7-27/9-3; habitat: rich flark fen; **Record Level:** institutionCode: JES
- **Type status:** Paratype. **Occurrence:** recordedBy: J. Salmela; individualCount: 1; sex: male; **Taxon:** genus: Neoplatyura; specificEpithet: noorae; scientificNameAuthorship: Salmela; **Location:** country: Finland; stateProvince: Lapponia kemensis pars occidentalis; verbatimLocality: Kittilä, Kielisenpalo; verbatimLatitude: 68.020; verbatimLongitude: 25.063; verbatimCoordinateSystem: decimal degrees; verbatimSRS: WGS84; **Event:** samplingProtocol: Malaise trap; eventDate: 2007-7-24/8-31; habitat: rich spring fen; **Record Level:** institutionCode: MZHF
- **Type status:** Other material. **Occurrence:** catalogNumber: MYCE-NV-2013-0077; recordedBy: J. Salmela; individualCount: 1; sex: male; **Taxon:** genus: Neoplatyura; specificEpithet: noorae; scientificNameAuthorship: Salmela; **Location:** country: Finland; stateProvince: Lapponia kemensis pars occidentalis; verbatimLocality: Kittilä, Nunaravuoma; verbatimLatitude: 67.699; verbatimLongitude: 25.353; verbatimCoordinateSystem: decimal degrees; verbatimSRS: WGS84; **Event:** samplingProtocol: Malaise trap; eventDate: 2007-6-27/7-24; habitat: poor sedge fen; **Record Level:** institutionCode: JES
- **Type status:** Other material. **Occurrence:** catalogNumber: MYCE-NV-2013-0101; recordedBy: J. Salmela; individualCount: 1; sex: male; **Taxon:** genus: Neoplatyura; specificEpithet: noorae; scientificNameAuthorship: Salmela; **Location:** country: Finland; stateProvince: Lapponia kemensis pars occidentalis; verbatimLocality: Kittilä, Kielisenpalo; verbatimLatitude: 68.020; verbatimLongitude: 25.063; verbatimCoordinateSystem: decimal degrees; verbatimSRS: WGS84; **Event:** samplingProtocol: Malaise trap; eventDate: 2007-6-26/7-24; habitat: rich spring fen; **Record Level:** institutionCode: JES
- **Type status:** Other material. **Occurrence:** catalogNumber: MYCE-NV-2013-0103; recordedBy: J. Salmela; individualCount: 2; sex: male; **Taxon:** genus: Neoplatyura; specificEpithet: noorae; scientificNameAuthorship: Salmela; **Location:** country: Finland; stateProvince: Lapponia kemensis pars occidentalis; verbatimLocality: Kittilä, Vuotsonperänjänkä; verbatimLatitude: 67.616; verbatimLongitude: 25.449; verbatimCoordinateSystem: decimal degrees; verbatimSRS: WGS84; **Event:** samplingProtocol: Malaise trap; eventDate: 2007-6-25/7-24; habitat: rich flark fen; **Record Level:** institutionCode: JES

#### Description

**Figure 2.**
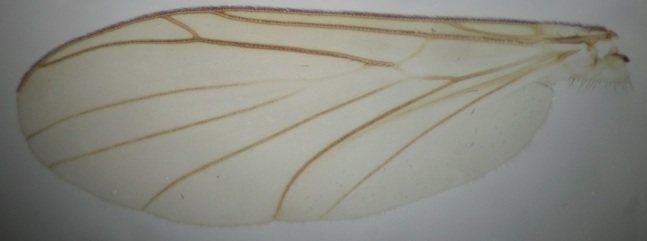
*Neoplatyura noorae* Salmela, sp. n., male, wing.

**Figure 3.**
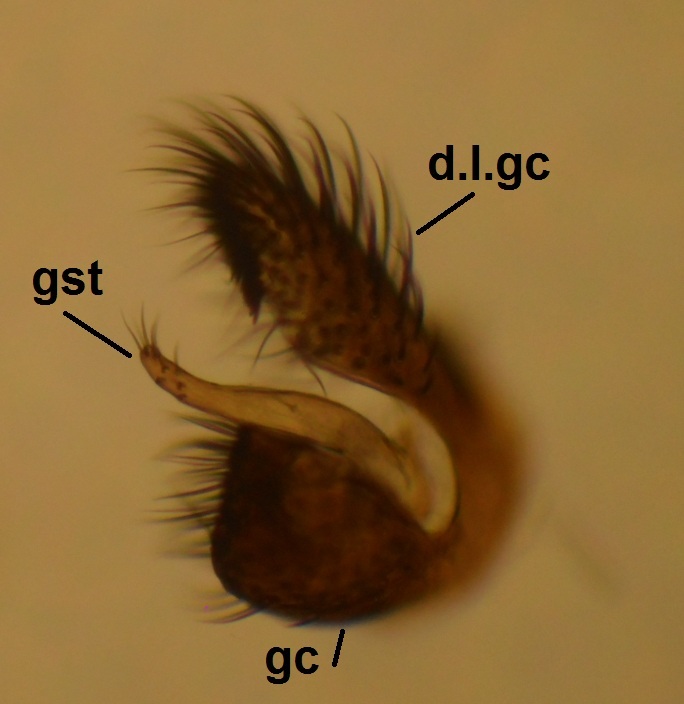
*Neoplatyura noorae* Salmela, sp. n., male gonocoxite and gonostylus, lateral view. Abbreviations: d.l.gc = dorsal lobe of gonocoxite, gst = gonostylus, gc = gonocoxite.

**Male** (n = 3 in measurements). **Head** dark brown (Fig. 1b). Three ocelli, bulging in lateral view, lateral ocelli not touching eye margins. Ommatidia pubescent. Antennae brown, 14-segmented. Scape length:width ratio 0.8–0.82, pedicel length:width ratio 0.82–0.85. Scape and pedicel with black setae, shorter than width of respective segment. First flagellomere elongated, 2.65–3.19 times longer than wide. Flagellomeres 2–13 shorter, ca. 1.05–1.4 times longer than wide, last segment length:width ratio 2–2.92. Flagellomeres bearing hyaline setae and flagellomeres 1–6 with some dark, stouter setae; five dark setae apically on last flagellomere. Palpus brown, 5-segmented (Fig. 1b). Palpus generally short, ca. as long as total length of three basal flagellomeres. Palpomeres 1–4 with stout dark setae, 1–3 per segment, third segment with a sensory pit. Last palpal segment 1.44–1.7 times longer than penultimate segment. Clypeus with 6–7 black setae. **Thorax** dark brown (Fig. 1b). Scutum, antepronotum and proepisternum with black setae. Scutellum with ca. 20 dark setae, arranged in a row. Coxae brown–light brown, bearing numerous black setae. Femora light brown, with black setae. Tibiae and tarsi light brown, bearing black setae, apically arranged in rows (rows are especially clear in mid and hind legs). Tibiae also with stronger black setae, increasing in number from fore to hind legs, shorter than respective tibial width. Mid and hind tibiae with apical combs, i.e. with a dense row of black setae. Spurs dark brown, spur formula 1:2:2. Fore leg: ratio of femur to tibia 0.85–0.89; ratio of tibia to basitarsus 1.18–1.22. Mid leg: ratio of femur to tibia 0.8–0.85; ratio of tibia to basitarsus 1.32–1.4. Hind leg: ratio of femur to tibia 0.68–0.7; ratio of tibia to basitarsus 1.53–1.6. Halteres light brown. **Wing** (Figs 1a, 2) pale brown, with no clouds or dark patterns, length 3.8-4 mm. Veins dark brown, but Rs, M stem and bases of M_1_ and M_2_ pale.  R_1_, R_5_, M_1_, M_2_, CuA_1_, CuA_2_ and A_2_ setulose above (bases of M_1_ and M_2_ bare). Veins r-m and R_5_ setulose ventrally. C not reaching middle to tips of R_5_ and M_1_. Sc ending in C before base of Rs. Setae present in wing cells a1 and a2. **Abdomen** dark brown, both tergites and sternites densely covered by dark setae. Gonocoxite pear-shaped, bearing long dark setae, and with glabrous median lobe (Figs 1c, 4a). Gonocoxite with dorsal lobe (Fig. 3), apically truncated and with a comb-like row of setae. Gonostylus finger-like, bearing setae especially in apical portion (Fig. 4a). Aedeagal complex ventrally with a granulated membrane; apex of that membrane with minute setae (Fig. 4a). Aedeagus bilobed, apices rounded and curved, basally with rather long anterior apodemes (Figs 1c, 4a). Proctiger isosceles trapezoidal (Fig. 4a), distal margin curved toward cerci. Cerci as in Fig. 4b.

**Female** unknown.

#### Diagnosis

Dark brown species with unpatterned wings. Apical setae of mid and hind tibiae arranged in rows. Gonocoxite pear-shaped with pronounced, glabrous median lobe. Gonocoxite with a conspicuous dorsal lobe, bearing a comb-like row of short and stout setae. Gonocoxites are widely separated ventrally while the other European species have them fused medially by a more or less wide bridge. Gonostylus finger-like. Based on unpatterned wings, body coloration and structure of male hypopygium, the species is easily distinguished from its congeners.

#### Etymology

The species is named after MSc Noora-Annukka Vartija, our friend and colleague. Noora Vartija sorted out and recognised this as a possible new species from large masses of Malaise trapped material from Finnish Lapland.

#### Distribution

European, so far only known from Finland. Most of the collecting sites are in central Lapland, north boreal ecoregion, in Kittilä municipality. There is also a record from southwestern Lapland, mid boreal ecoregion, Tornio municipality.

#### Ecology

Invariably, all collecting sites are pristine boreal peatlands. The collecting localities are minetrophic fens, mostly open or sparsely wooded. Most of the sites are wet rich fens, lying on calcareous bedrock, being characterized by brown mosses (e.g. *Scorpidium*, *Paludella*). Only one of the sites is a poor sedge fen, dominated by *Sphagnum* mosses in the ground layer. The species has been collected with fungus gnats such as *Mycomya fennica* Väisänen, *Boletina dubia* Meigen, *Boletina intermedia* Lundström, *Sciophila bicuspidata* Zaitzev, *Acnemia trifida* Zaitzev and *Isoneuromyia semirufa* Meigen. Larval microhabitat is unknown.

#### Conservation

Six out of seven collecting sites are conservation areas, protected by Nature Conservation Act. The species seems to have restricted occurrence in Finland, both geographically (north Finland) and ecologically (pristine fens), and could perhaps be later classified as a threatened species following IUCN criteria (see e.g. Penttinen et al. 2010).

#### Taxon discussion

The species is readily distingushed from its European congeners. If added to the key provided by Hutson et al. 1980 (p.37) *Neoplatyura noorae* Salmela, sp. n. would be keyed out in the first couplet, because it has unpatterned wings (*Neoplatyurabiumbrata* (Edwards) with darkened wing tip and a cloud on posterior vein of CuA_2_) and being dark brown species (the rest of the European species are yellowish). Nearctic species *Neoplatyura pullata* (Coquillett) is a dark species, but has Sc ending in C at the level Rs (in *Neoplatyura noorae* Sc ending in C before Rs).  It should be noted that *Neoplatyura pullata* is only known from a female holotype specimen, collected from USA, California (Johannsen 1910, Evenhuis 2006).

## Supplementary Material

XML Treatment for Neoplatyura noorae

## Figures and Tables

**Figure 1a.**
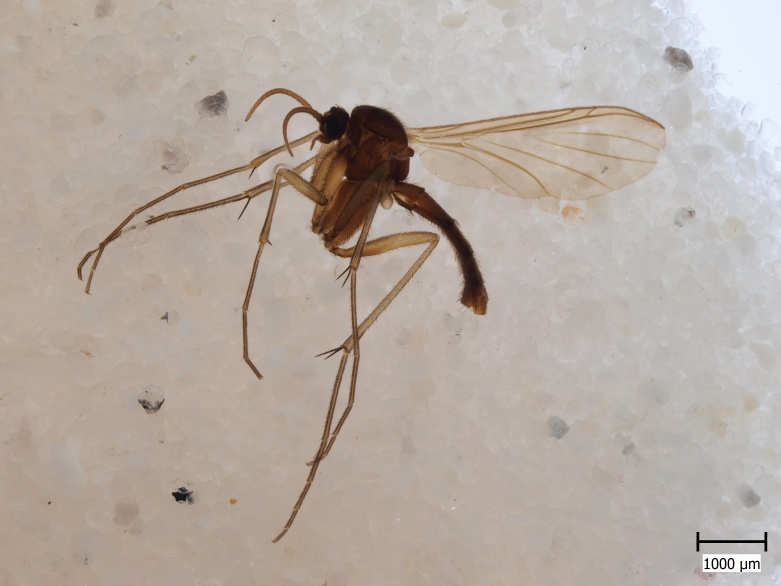
Habitus, lateral view. Hypopygium detached.

**Figure 1b.**
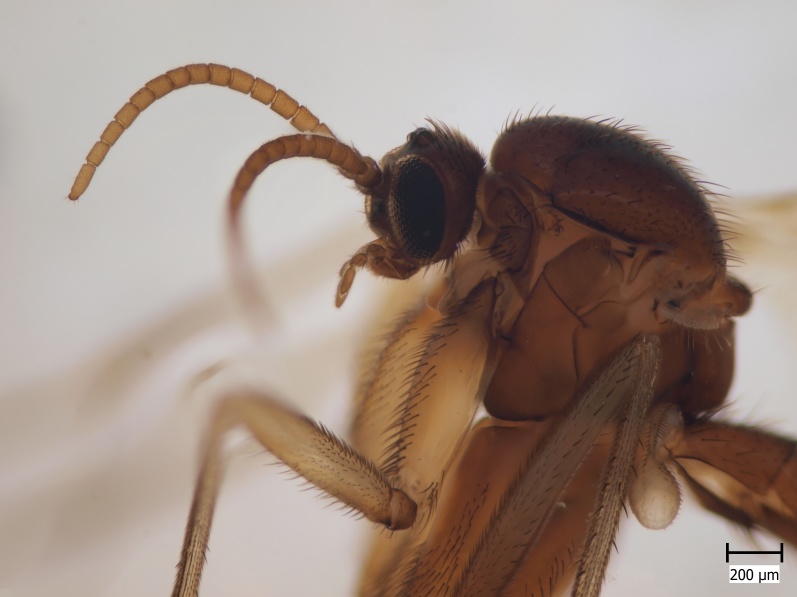
Head and thorax, lateral view.

**Figure 1c.**
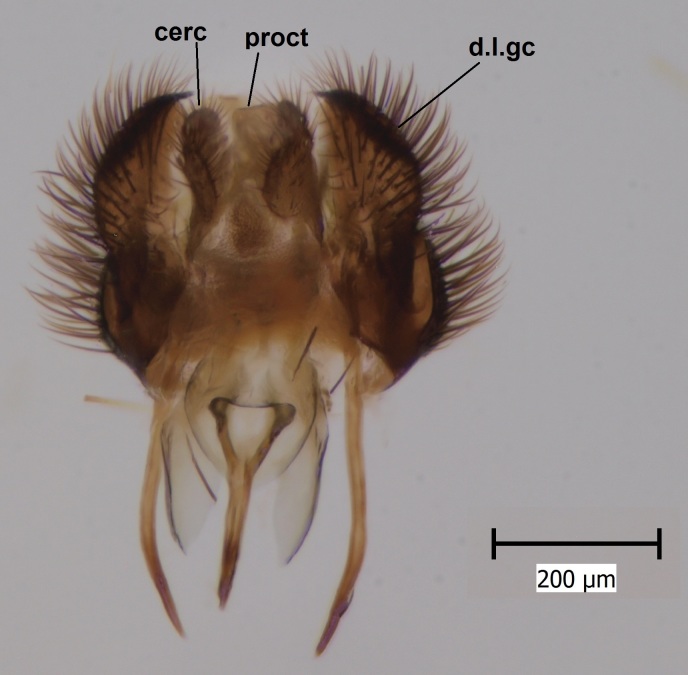
Hypopygium, dorsal view. Abbreviations: cerc = cerci, proct = proctiger, d.l.gc = dorsal lobe of gonocoxite.

**Figure 1d.**
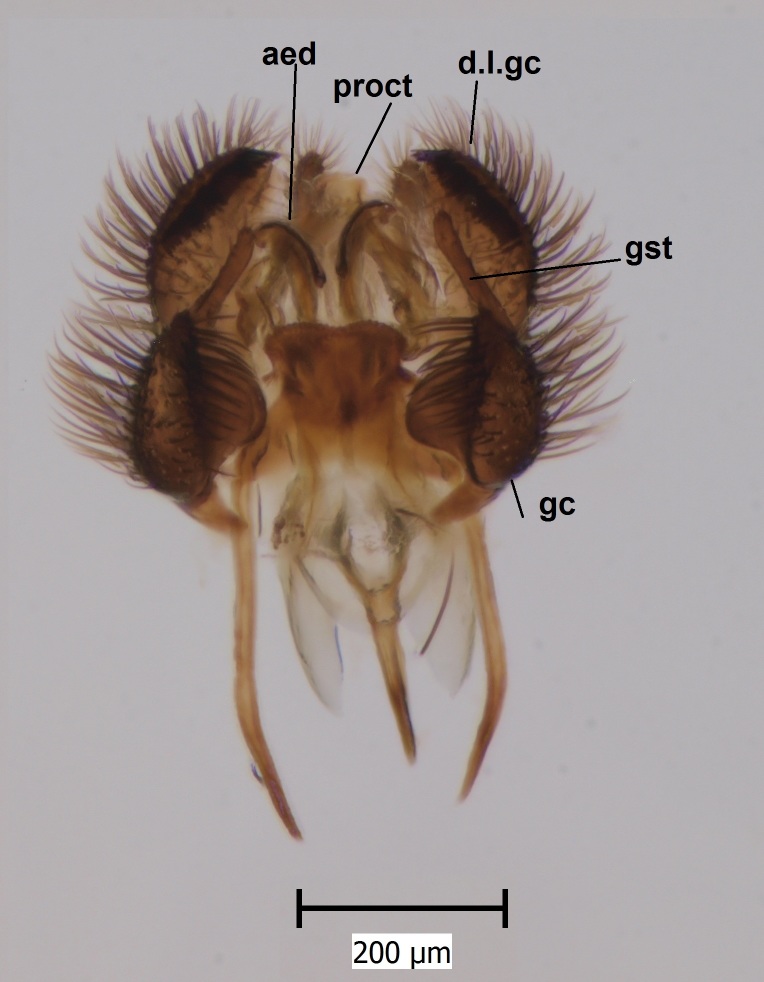
Hypopygium, ventral view. Abbreviations: aed = aedeagus, d.l.gc = dorsal lobe of gonocoxite, gst = gonostylus, gx = gonocoxite, proct = proctiger.

**Figure 4a.**
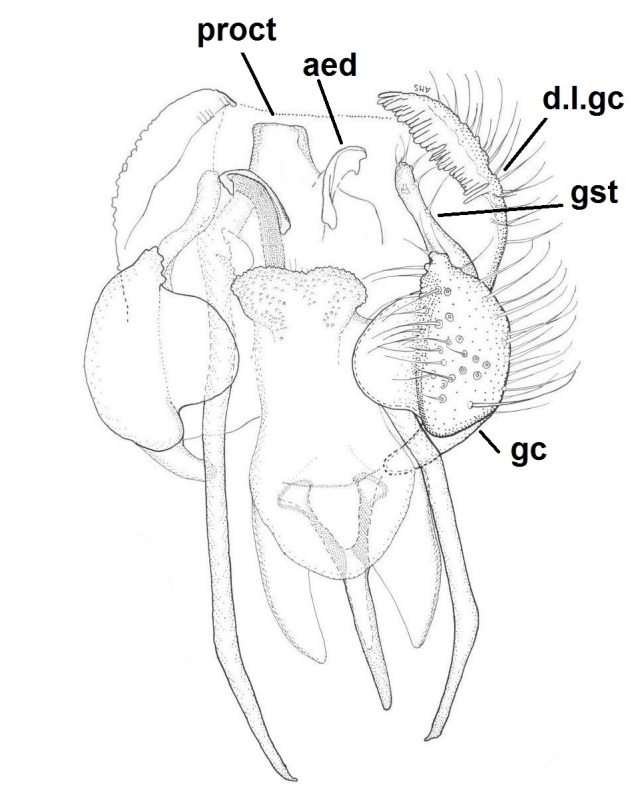
Dorsal view. Abbreviations: proct = proctiger, aed = aedeagus, d.l.gc = dorsal lobe of gonocoxite, gst = gonostylus, gc = gonocoxite.

**Figure 4b.**
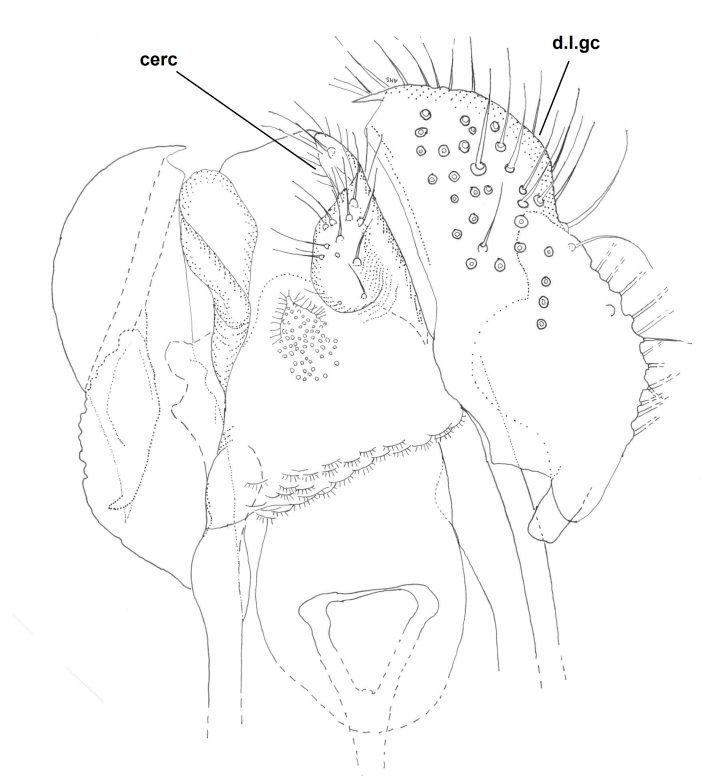
Ventral view. Abbreviations: cerc = cerci, d.l.gc = dorsal lobe of gonocoxite.
